# Gynecological cancer patients’ differentiated use of help from a nurse navigator: a qualitative study

**DOI:** 10.1186/1472-6963-12-168

**Published:** 2012-06-21

**Authors:** Marianne K Thygesen, Birthe D Pedersen, Jakob Kragstrup, Lis Wagner, Ole Mogensen

**Affiliations:** 1 Department of Gynecology and Obstetrics, Odense University Hospital, Institute of Clinical Research, Faculty of Health Sciences, University of Southern Denmark, Sdr. Boulevard, Odense, Denmark; 2 Research Unit of Nursing, Institute of Clinical Research, Faculty of Health Sciences, University of Southern Denmark, Campusvej, Odense, Denmark; 3 Research Unit of General Practice, Institute of Public Health, Faculty of Health Sciences, University of Southern Denmark, J.B.Winsloewsvej, Odense, Denmark

**Keywords:** Nurse navigator, Patients’ view, Distrust, Qualitative research

## Abstract

**Background:**

Fragmentation in healthcare can present challenges for patients with suspected cancer. It can add to existing anxiety, fear, despair and confusion during disease trajectory. In some circumstances patients are offered help from an extra contact person, a Nurse Navigator (NN). Scientific studies showing who will benefit from the extra help offered are missing. This study aims to explore who could benefit from the help on offer from a nurse appointed as NN in the early part of a cancer trajectory, and what would be meaningful experiences in this context.

**Methods:**

A longitudinal study with a basis in phenomenology and hermeneutics was performed among Danish women with gynecological cancer. Semi-structured interviews provided data for the analysis, and comprehensive understanding was arrived at by first adopting an open-minded approach to the transcripts and by working at three analytical levels.

**Results:**

Prior experience of trust, guarded trust or distrust of physicians in advance of encountering the NN was of importance in determining whether or not to accept help from the NN. For those lacking trust in physicians and without a close relationship to a healthcare professional, the NN offered a new trusting relationship and they felt reassured by her help.

**Conclusions:**

Not everyone could use the help offered by the NN. This knowledge is vital both to healthcare practitioners and to administrators, who want to do their best for cancer patients but who are obliged to consider financial consequences. Moreover patients’ guarded trust or distrust in physicians established prior to meeting the NN showed possible importance for choosing extra help from the NN. These findings suggest increased focus on patients’ trust in healthcare professionals. How to find the most reliable method to identify those who can use the help is still a question for further debate and research.

## Background

The cancer journey can present a frightening and stressful challenge [1,2]. Uncertainty, worries and anxiety are experienced throughout the disease trajectory [2-4]. Furthermore, undergoing specialized treatment in healthcare can cause patients’ to feel that they are being delayed [1] and from the healthcare professionals’ point of view the fragmentation of care seems to contribute to patients feeling overwhelmed [5,6]. Surveys from the UK and Denmark indicate that some patients with cancer might wish for more help in these areas [7-9], but we lack scientific studies showing who can make use of the extra help offered.

Many western countries tackle these problems by offering an extra person to support and guide patients from the time of suspicion of cancer, or from the time of diagnosis [1,5,6,10-12]. In the US the focus for such care has been on low-income groups, as they are found to be most likely to suffer delay [12], but there is a tendency to focus increasingly on social and psychological support [13] and to extend the offer of help to more or all cancer patients [5,6,14-17]. Research in the Nordic countries has proposed that the offer of support should be aimed at all patients with cancer [1,18], and in Denmark a healthcare professional contact person is required by law [19,20]. Thus, there is no consensus as to whether it is all [1,18,19] or some patients with cancer [15,21-24] who should be targeted for additional help, or what kind of additional help the patients should have. Navigators in cancer care have been proposed to embrace this extra help. They help the cancer patient “not only travel the healthcare maze in a more timely fashion, but [the patient’s] psychosocial well-being and quality of life may also be enhanced” [25] p. 29. Navigation is offered to patients for a predefined period, which can differ from setting to setting. The job includes identification, planning, and following patients with regard to individual barriers to receiving care. This could induce coordination, linking to additional help, and emotional support, as well as help to educate patients to manage on their own [12]. It can for instance be done by a lay community peer, a medical assistant, a social worker or a cancer survivor with minor courses in healthcare [5,12]. It can also be done by a nurse (a Nurse Navigator (NN)) where post holders, moreover, give tailored disease- and illness-specific information [16], including explanation of information given earlier by physicians. Among the multiple roles of supplementary assistants to the healthcare system Case Management is probably the best known role [26]. This role has been used in the US for decades to minimize cost and help patients through a part of a disease trajectory. Within nursing, Case Management has been developed and can be divided into three generations [27]. As a third generation Case Manager, the NN’s approach to patients is holistic and empowering, and extra help is offered in transitions in the healthcare system [14,27]. On the other hand, a difference in the contact to the patients can be present. Where a Case Manager seems to be responsible for an open line of communication with patients, and in this way being the one, who takes initiative to contact to the patients [28,29], the NN seems to have the same or a somewhat minor proactive role *and* a focus on availability [5,14-17,21]. Moreover, where a healthcare professional contact person at most offers coordination, continued contact and conversation at the hospital, an NN’s offer of help reaches out in the period the patients are not at the hospital where the NN is available with help on phone. This study aims to explore who could make use of the help offered from a nurse appointed as an NN in the early part of a cancer trajectory, and what patients experienced to be meaningful in this connection. Here the early part considered to be the diagnostic phase of the disease and while waiting for primary cancer treatment.

## Methods

A qualitative study was found appropriate as experiences and meanings were desired, and limited knowledge of this area of study is available [30]. The study used a phenomenological- hermeneutical approach.

### Setting

The study took place in Denmark, where the healthcare system is primarily financed through taxes and is free for all citizens [31]. People are in this way confident of getting help. As our starting point we used a gynecological surgical unit at Odense University Hospital, as the cancer patients at this unit were offered an NN in a pilot scheme. The unit receives patients from the entire Region of Southern Denmark (1.2 million inhabitants).

### Nurse navigator (NN)

The NN was available for contact during all office hours from the day after the patient’s referral had been received by the outpatient clinic and until subsequent referral or hospitalization in connection to the clinic. The NN was female, and offered help in the area of coordination and information. Moreover, she had time to listen to the patients’ worries and could give advice with regard to facing the situation with cancer. She could support the development of a local resource network, and link to other resources if necessary. Moreover, the NN advocated for the patients at the hospital. She updated the physicians at the outpatient clinic with information about the patient, thereby optimizing the physicians’ individual information to the patient. This kind of help was regarded as additional help. The nurse appointed as the NN also had a job as nurse in the outpatient setting, where she accompanied the patient to the appointment with the physician, and afterwards followed up with regard to information on the disease and the treatment plan, and answered questions from the patient and possible accompanying relative.

The NN was introduced to the patient as a person with considerable experience of oncological nursing and would address the patient’s prioritized concerns, as well as provide the scheduled times for examination and for any visit to the outpatient clinic. The NN would rearrange these appointments, if they did not suit the patient. Moreover, the patient was assured she could contact the NN during the available period, and would meet the NN at the outpatient clinic. The patient was promised that she would also receive the scheduled times in the mail. All patients were provided with the NN’s phone number twice; both by mail and telephone.

### Participants

**Figure 1:**
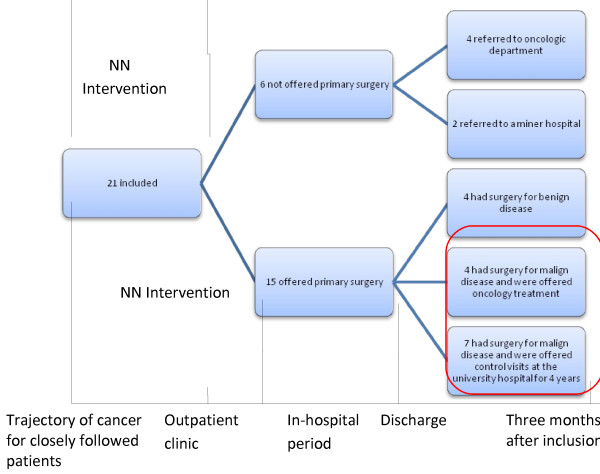
**Flowchart and trajectory.** The red line marks the more closely followed groups. NN: Period with an available Nurse Navigator.

A total of 30 women were contacted consecutively by phone and invited to take part in the study. The inclusion criteria were that a patient should be suspected of having gynecological cancer and able to speak and understand Danish. No exclusions were made because diversities were desired among the participants. Nine declined to participate because they did not wish to focus on the issue of their cancer (n = 3), felt lack of energy (n = 3) or simply did not want to spend time on it (n = 3). These were slightly younger (median age 55 (range 32 – 79)) than those who participated. We included 21 participants with a range of characteristics including age (median age 63 (range 36–79)), marital status, place of residence and gynecological diagnosis (Table 1). All characteristics in Table 1 emerged in the process of data collection, and are presented here to strengthen reporting with “thick contextual descriptions” [30] p 55*.* Only eleven underwent primary surgery to remove cancer. These eleven participants were selected for closer study, since they as participants with cancer would have the maximum period of help offered by the NN, namely from inclusion in the study through to the in-hospital period (Figure 1). These eleven presented same diversity in characteristics as shown in Table 1.

**Table 1:** Differences in basic socio-demographic characteristics and illness- and disease specific characteristics

**N**		**1**	**2**	**3**	**4**	**5**	**6**	**7**	**8**	**9**	**10**	**11**	**12**	**13**	**14**	**15**	**16**	**17**	**18**	**19**	**20**	**21**
**Age**		**76**	**65**	**56**	**43**	**37**	**68**	**63**	**70**	**37**	**79**	**54**	**66**	**61**	**59**	**79**	**36**	**46**	**55**	**69**	**70**	**71**
Marital status	Live with partner most days	x	x	X	x	x	x	x	x	x			x	x	x		x		x			x
	Live with partner a few days a week										x	x				x						
	Live alone																x			x		
Place of residence	Town	x	x	X	x	x			x	x			x	x	x		x		x	x		x
	Countryside						x	x			x	x				x		x			x	
Diagnostic phase	Diagnosed		x			x			x		x		x		x		x		x		x	x
	Diagnostic phase	x		x	x		x	x		x		x		x		x		x		x		
Diagnosis (suspicion of)	Ovarian cancer	x	x	x			x		x			x	x		x	x				x		
	Uterine cancer							x			x			x					x		x	x
	Cervical cancer				x	x				x							x	x				

### Data

Narratives obtained through semi-structured interviews were chosen as the main data source as this is a good way of gaining insight into the world as experienced by the participants [30]. In order to support the interviews, participants completed pre-printed diaries to hold on to experiences and to give the reader insight into the trajectory as experienced by the participants [32,33]. All participants completed diaries in the period they were offered help by the NN. The more closely followed had diaries until end of the study (Table 2). Moreover, the interviewer made observations at the outpatient clinic (if patients consented) prior to the interviews. Data from completed diaries and observations provided information about the individual person, and increased the possibility of asking meaningful questions in the interviews, and were only used as such. Since it might help persons to write down negative emotions in a coherent narrative [34], and since this might interfere with the use of additional help from the NN, the diary had a semistructured design that only left a quarter of an A4 sheet for an extended account of feelings and emotions. Before a specific interview the available diary (diaries) and notes from the observations in relation to the specific participant were read. All interviews were conducted by the first author. Interviews were supported by a general semi-structured interview guide and a visual elicitation technique, which was developed during the study and which helped participants to remember and to create deeper narratives while drawing graphs [35]. The interview guide included themes and suggestions for open-ended prompt questions [30] such as “What was your experience of suspicion being aroused that you had cancer? “What was your experience of the trajectory?” and “How have you made use of healthcare professionals?” Moreover, the patients were asked about their experiences with the healthcare persons, who the interviewer was aware of had been a part of the patient’s trajectory of disease. Participants decided where to be interviewed as it was important that they felt comfortable. For those participants who did not choose their home, a room at the hospital was provided where they could talk confidentially and undisturbed.

**Table 2:** Data sources

**Trajectory**	**Outpatient**	**In-hospital**	**Outpatient**
**Interviews**		(Interview at discharge)	Interview 3 months
			after inclusion
**Background data**	Observation		
**for interview**	Diary	(Diary)	(Diary)

() marks the extra data sources for the special followed.

For all participants, interviews were conducted approximately three months after their enrolment in the study. This was done in order to establish a distance from the experiences of (possible) acute cancer but not so great a distance from the experiences with the NN that these had been forgotten. At discharge one additional interview was conducted with those participants who were studied more closely in order to hold on to their experiences (Table 2). In this way the help offered by the NN for these participants was talked through in two interviews. All interviews lasted on average one hour and were recorded and transcribed ad verbatim.

### Analysis

An interpretative method of phenomenological hermeneutics based on Ricoeur’s theory of interpretation [36] was chosen. This theory has inspired development and the use of interpretative methods in research in the Nordic countries since the 1990s [37-39]. When taking this approach it is not the objective to “guess” the participants’ intentions with what is narrated, but instead to present what the text (the narratives) opens for, between the text and the interpreter [36] p. 152, [38] p. 70: The analysis of the narratives was carried out at three levels. The first level is an overall first very broad interpretation labelled “naive reading”. At the second level a non-thematic structural analysis is performed by approaching the text in an open-minded way and disclosing units of meaning (what was said) and units of significance (what the text talks about), which was subsequently collated in themes. The themes were reflected upon to see if they could substantiate and validate the naive understanding, which was subsequently more precisely formulated. At the third level, a comprehensive understanding and discussion were carried out. In this process literature relevant for the topics, as well as parts of the narratives, was included to widen the interpretation further. The first author undertook the analysis, but interim results were scrutinized and discussed between authors on several occasions during this process. Nvivo7 was used as support in the analysis.

### Ethics

The participants were verbally informed about the study and were invited to ask questions about the study before agreeing to participate, and again before their outpatient visit, when informed content was signed. It was important that the participant was feeling comfortable, and the method was designed in a way which allowed first author to give distressed participants space, for instance by offering more time, and accepting smaller narratives. In order to maintain anonymity no names are used, and all physicians are described as male. The study adheres to the Declaration of Helsinki [40] and Ethical Guidelines for Nursing Research in the Nordic Countries [41]. The Biomedical Research Committee System Act at the Scientific Ethics Committee for the Region of Southern Denmark does not apply to this project. The Danish Data Protection Agency gave formal consent to the study (CVR-nr. 11-88-37-29).

## Results

A naive reading and structural analysis of the data pointed at trust or guarded trust (or even distrust) of healthcare professionals as a reason that contributed to whether the patient could use additional help from the NN. However, a “closely related healthcare professional” (a physician, a nurse, a psychologist, a medical secretary among their loved ones who was not a part of the hospital) seemed to be the joker. In the following we outline and elaborate on the central findings in the structural analysis. The quotations are presented including dots (…) indicating that text is left out to condense a message, square brackets are used where the author puts condensing words on a participant’s longer part of narrations, and where the interviewer (and first author) interact in words with the participant, this is reported in round brackets.

The essence of the narratives did not change over the two interviews with regard to the aim of the paper, and the quotations are chosen where a participant most precisely explained the situation as it was experienced.

### Trust in healthcare professionals

Some participants expected caring and calm healthcare professionals who could explain matters in a way they could understand, and they expected to be taken seriously. These expectations were fulfilled by some participants in the period prior to meeting the NN. They had the information they needed and felt that adequate care included having dates for examinations and date for an outpatient visit posted to them and in this way they accepted a wait of one or two days before getting these dates. They trusted physicians and other healthcare professionals, and declined the offer of extra help from the NN, which is outlined in the following quote:

"*Physicians themselves are calm and explain…he did not make me [totally confused]… so…it was not necessary [to use the NN]. All the time I felt they were very caring and I had confidence in the situation…I will talk to my good general practitioner later.*"

"*(At discharge)*"

Expected and experienced care from calm health professionals were factors that together meant that additional help from the NN was declined. Should these participants encounter problems later, they would talk to their general practitioner whom they trusted would help them. The NN offered a supportive talk, and some of the participants who trusted physicians had listened to the NN. They had shown no interest in further contact with the NN, and received the dates for appointments at the hospital, but did not write them down, and could not repeat them to their close relatives. Others had listened to and talked a little with the NN in what they estimated to be up to twenty minutes. They all found her to be a nice person and regarded the offer as positive overall, but had not the heart to tell the NN that the extra help was not useful to them. These participants showed differences in ages, marital status, place of residence and experienced diagnostic phase as well as (suspicion of) diagnosis (Table 1). Furthermore, some were financially well-off which gave them the possibility of buying recreational experiences, so that thoughts of cancer before start of treatment could be diverted. Other participants have experienced a tight financial situation and tried to free themselves from thoughts of cancer by intensifying work, visiting friends or increasing the focus on their children. Time from first symptom to contact to the NN differed by more than a month, and to some patients healthcare professionals were very great authorities. Moreover, some had experienced a bad situation with nurses before talking to the NN. This was a picture mirrored as well in all the following reported “groups”. Some of the participants, who had trust in physicians, also had close relations to a healthcare professional (family or friend), hereafter labeled “closely related healthcare professionals”.

### Trust in a closely related healthcare professional

All participants had one or two among those closest to them to talk to about the changed situation brought on by cancer, but some participants had close relations to a healthcare worker who helped them in the same area as the NN was offering the additional help. These “closely related healthcare professionals” were central, and the participants felt deeply obligated to them and never questioned their words or intentions. These participants did not use the extra help from the NN. They could have trust in physicians, as put forth in the following quotes..:

"*I have not used [the NN] because I have my children…[one of whom is a healthcare professional and] she has followed me through this. (At discharge)*"

"*[The “closely related healthcare professional”] has been a good girl…When…I had any questions she immediately went to the computer…[This made me] comfortable (I:mm)…I trusted the doctors… I did exactly as the [they] said. (Three months after enrolment in the study)*"

.. or they could have bad experiences with physicians prior to meeting the NN, which in their narratives were followed by generalization. Such bad experiences are reflected in the following quote, where a participant at the local hospital had the same investigations ordered twice and refused to show up to the same investigations again:

"*It ended in a complete mess and if others experience what I did (I: yes) then they are just a bunch of lousy patients who don’t bother to come when they’re supposed to.*"

"*I never talked to [the NN]…instead…Mostly I have talked to my good [“closely related healthcare professional”] (I: yes) when…I had a question…she knows nearly as much as the others in the system.*"

"*(Three months after enrolment in the study)*"

Regardless of whatever these participants felt they received a good medical treatment, information and guidance, and felt trust in physicians, or felt the physicians were not in control of the diagnostic process, and generalized negative experiences, these participants did not use the extra help offered by the NN, because they each had a “closely related healthcare professional”. Participants had frequent contact with these private helpers, in certain periods every day for emotional talk or for help in one of the other named categories of help also offered by the NN. These participants briefly listened to the NN when she called, and although they could not use the help, some found her nice to talk to. A call from the NN was accepted by all but one. Moreover, a participant was not totally aware of the suspicion of cancer, and from her it was questioned, if an NN was the right person to call patients and give information about more investigations. This participant did not find the NN nice to talk to, as she brought bad information, but on the other hand the participant found the NN to be a decent and knowledgeable person. In this way the NN function was questioned, not the NN person.

However, this group of participants trusted a “close healthcare professional” and used this person in preference to the NN, regardless of any trust or distrust in a physician they had encountered in a disease trajectory before meeting the NN.

### Distrust (or guarded trust) in healthcare professionals

Some participants could use the additional help offered by the NN. Although some of these participants had negative experiences with all kinds of healthcare professionals, negative experiences with physicians prior to meeting the NN seemed important. These negative experiences were all spontaneous parts of the narratives, and included a physician they considered important in a sickness trajectory after a hospitalization. The general practitioner could be regarded as non-important in a hospital context, and with lack of knowledge after discharge, and in these cases earlier experiences with hospital physicians were important. These experiences were narrated as latest nonverbal experience with such a physician before the contact with the NN, and were sometimes, and sometimes not, accompanied by moral condemnation of physicians or of the whole hospital sector, like in the following quotes:

"*Then Circus Hospital Authority starts up… nothing ever goes as the way it should and no times that fit…hope they don’t fall over each other…It’s all very well the doctor being busy…but I bloody well think it’s not good enough that he doesn’t call and say…if you need me, I’m right here. I have heard nothing. I find that scary. (At discharge)*"

"*Doctors… come and go as they see fit… they come and say their bit and then they leave again (yes) they can go as far as to turn their back on you at the same time, I mean . . really! . . (I: Has anybody done that?). No, I don’t think [doctors at the hospital] did, but I have seen others previously.*"

"*(Three months after enrolment in the study)*"

Condemnations included verbal and non-verbal signals of healthcare persons who in general lack seriousness and of physicians who cannot be relied upon. An unpleasant experience of not having been taken seriously by physicians took place either in a prolonged diagnostic phase or in an earlier period in hospital. In such a period a participant e.g. encountered a physician who did not treat her and a fellow patient in a correct way by “just turning his back on them” in conversation, and another participant felt a hurtful lack of interest from her primary physician after this physician was told the participant had cancer. These participants had given up expectation of a hospital sector that was coherent and effective, or physicians they could trust. They had built up guarded trust (or even distrust) in physicians and in the hospital system as a whole before meeting the NN. Despite this, the NN was found to be a nice person.

### Trust in the NN

Those who could use the extra help from the NN found her “very kind” and calming, as reflected in the following quote:

"*The NN was, in fact, quite nice … she was able to tell me more … and she explained things, so I really trusted her (ok) … it was before treatment … and everything was uncertain (yes), so I got the opportunity to ask some questions (ok), and I actually remember that she said that she had 25 years of experience; she had seen some things, and they would result in such and such … and that I should take it easy and so on (yes) … and I was much relieved (yes), really … [the NN] had a professional attitude …*"

"*(Three months after enrolment in the study)*"

The way the NN acted mirrored what patients expected of the healthcare professionals and the participants trusted her.

The NN represented these participants’ interests, and help from her was accepted. Although all participants described experiences of periods with negative emotions filling their thoughts, this was more salient by those who gained trust in the NN, and they all felt calmed by her.

## Comprehensive understanding and discussion

This article provides insight into the experiences of cancer patients with regard to help offered in the early part of the cancer trajectory by a female nurse navigator (NN).

When participants had the opportunity to enlist extra help from the NN, the majority of the participants did so, but not all, although all participants found her nice and welcoming or a decent and knowledgeable person. On the other hand it seemed all could take advantage of the feeling of trust in a healthcare professional in the early part of a cancer trajectory. If a participant did not have this sense of trust with regard to the physicians *or* did not have a helping healthcare professional among her close relations *and* did not feel sure such a specific healthcare professional would help her, she could use an obliging healthcare contact person (an NN), who would help her address whatever problem she might encounter.

### Trust – guarded trust - distrust

Although our study showed that trust in healthcare professionals was important for patients, we also found that distrust could be present. Narratives from our study included bad experiences with physicians, often including moral condemnations generalized to the whole hospital system. In such cases the NN was used. It can be argued that the NN was a part of the hospital system and in this way it might be surprising that the NN as an extra person was allowed to help. The theory of trust and distrust, put forward by the Danish philosopher Løgstrup [42], seems helpful in explaining why. If one is deeply disappointed by another person’s way of acting in a non-customary way, one loses faith in the other and can morally condemn the other or even all of his kind, but this position can be reversible. If the disappointed person had not “closed himself off and acted in coldness” [42] p. 25 he can display guarded trust. In such situations the trust can be recreated if the other person does what the disappointed person experiences as customary 42]. In our study the NN was by all participants found to be a nice or decent person and despite widely generalized distrust, it was therefore possible to build trust in her.

Our findings suggest lack of patient trust in healthcare professionals as brought about by patients interpreting healthcare professionals’ signals as not conforming to what they perceive as customary, which is also indicated in other research [43-45]. In the UK Brown investigated gynecological cancer patients’ approach to the healthcare system in a situation with medical errors brought forward in the media and found more levels of experience relevant for trust; the abstract, the public and the private, where the first level is not linked to direct experiences but manifested through the media. The other two levels concern the direct experiences based on observations and the inter-subjective experience and these two levels were found to be very important for the patients’ experiences [45]. Irrespective of whatever the starting point is trust as something present and fundamental from the very beginning, as reflected in Løgstrup’s theory [42], or whether it is something that must be built up, as for instance reflected in the work of Brown, justifying his approach in the work of Schultz [45], the experience of the interaction between human beings is found to be crucial for the feeling of trust [42,45]. In our study the participants, who could not use the additional help from the NN, described a trusting relationship with a special and for many a long-known healthcare person and this relationship was given as a reason for not using the extra help from the NN. The interactions with these healthcare persons were described as being quite similar to those other participants had with the NN; they were available, they helped and were found to be very nice persons. Løgstrup’s theory of trust and distrust emphasizes the fact that we trust the other to do what we regard as customary, and if they do, they retain our trust. Otherwise we create guarded trust or even distrust [42]. Using Løgstrup’s theory of trust and distrust, it could be argued that the healthcare professionals, who by the participants in our study were allowed to help, understood and interacted within the acceptable in relation to these participants’ moral concepts. They acted as expected with regard to competences and non-verbal signals. In this way both the NN, the trusted physicians, and the healthcare professionals who were closely related to some participants were considered to be culturally competent to help the specific participants, and the long relation was preferred. These findings are supported by the research by Brown, who emphasizes the patient as the interpreter using moral judgement, and furthermore, he found patients could gain trust in healthcare professionals, if they had “mutuality of accounts” [45] p. 400 with the patients. This kind of cultural competence of healthcare professionals to secure trustful patients is also found useful within other research of navigation programmes. Laypersons, from the same culture as the patients, learn about the cancer patients’ disease trajectory in the healthcare system and help patients overcome barriers to healthcare [12,46,47]. In this way the close healthcare professionals in our study were close to such a lay person role. A grant from the National Cancer Institute in the US has ensured in-depth research with regard to such navigation programmes [12]. Exactly cultural homophily and community sensitivity were found to be important features in lay person navigatores [47], features able to remove barriers to proper health care for underserved breast cancer patients in the US [46]. Distrust in healthcare staff as well as experiences of disrespectful healthcare staff were some of the barriers reported by patients who could use the extra help [46]. With tasks additional to the “existing” system, and as a culturally sensitive healthcare professional, the NN in our research could also help some female patients, who gained trust in the NN, but a longer relation was preferred, possible because patients may experience a kind of connectedness [48,49].

Another finding in our research was the importance for the patients to have a trusting relationship with a physician they had met before they were referred to the hospital. We found most salient features of negative emotions before meeting the NN, in those who could use the additional help from the NN, which were the ones who had guarded trust or even distrust in physicians. This is in line with earlier findings suggesting experienced trust in physicians of importance to patients’ experienced mental quality of life [50]. As in our research Brown found trust in physicians of special importance for female patients with gynecological cancer [45]. Considering the fact that physicians are the ones expected to make diagnosis and treatment of diseases, it is possible trust in physicians more generally is important for female cancer patients.

### Use of an NN

It is worth noting, that not all patients awaiting diagnosis of possible cancer or primary treatment for cancer could take advantage of extra help from an NN. Similar results are reported from studies, where an NN was offered to patients with breast cancer or head and neck cancer [15,21]. Moreover, this is in line with research on lay person navigation and with the Institute of Medicine’s advice to the US government [22,23,51]. In 2008 they set up a standard for psychosocial healthcare to cancer patients, a standard that could be met by using an NN to make need assessments, link appropriate services to patients and make a subsequent follow-up and evaluation. Their advice was that all cancer patients in the US should have the psychosocial care they need, but also they noted that it was not certain that all patients with cancer could use extra help [22] (p. 199).

There is a tendency to consider a healthcare person as contact person in the care for patients in the early part of the trajectory of cancer [1,18,19]. The NN in this study also had such a function, but not all could use her extra help. This is in line with expectations in a qualitative interview study from the UK by Sinfield et al. concerning experiences of care recorded by men with prostate cancer [24]. It is, however, not what is suggested from grounded theory studies of this period of a trajectory of disease [1,19]. On the contrary, such studies propose that as a minimum there should be a nurse as special contact person for all patients with cancer. Giske et al. [18] have explored the experience and handling of Norwegian gastrointestinal patients while awaiting diagnosis in a hospital setting. The participants in the study were in an outpatient setting, which could make a difference to these findings, but it is worth noting that in the analysis Giske et al. eliminated data concerning “patients’ experiences and strategies related to staff” [18] p. 24, which was the kind of data we analyzed and which showed that not everybody needs a professional healthcare contact person. Jakobsson et al. [1] investigated experiences of newly diagnosed Swedish cancer patients in conjunction with a first outpatient appointment, where oncological treatment after surgery was a theme. They found that patients’ feelings of confidence was an important factor in achieving acceptance of their situation and pointing to the importance of the way in which healthcare professionals offer help. For further research they propose investigating whether it would help having a nurse maintain contact with patients while they wait for treatment. The nurse should provide help to meet the patients’ needs found in the above study, namely individualized timely care delivered with an element of person continuity. In our setting with patients awaiting cancer surgery, we found that it was not all patients who could use the additional help offered by the nurse (the NN), but only those who lacked trust in the physicians and had no healthcare professional among those closest to them.

### Strengths and limitations of the study

A weakness of this study is that the research project in itself might have represented an intervention and reduced the use made of the NN, since it might help persons to write down negative emotions in a coherent narrative [34]. Since all participants wrote diaries, all had the possibility of getting help this way. Because of this the diary had a semi-structured design that only left a quarter of an A4 sheet for an extended account of feelings and emotions. It is possible some participants would have acted a little differently*,* if the diaries were not handed to them, but not many lines were written in this section, and in this way reduced this limitation. A strength of this study is the longitudinal study design with techniques that a) helped participants to remember and in this way increased inclusion in the narratives of what we were interested in knowledge about, and b) provided a body of collective text which was used as a base in the interviews, one of these techniques being diaries, and c) positioned the interviewer as a not total stranger. These conditions were all counted on to give more elaborated narratives from the participants. Three months after inclusion all participants in this study had, among others, been informed about the seriousness of their disease, which could have influenced their narratives. Earlier experiences are always narrated from the present situation, and always from an interpreted world [52] p. 38-41. Because of this, the diaries were also used to support the memory, as was the cause for prioritizing the additional interview at discharge with the more closely followed, where many did not know either their diagnosis for certain or the further plan. These participants’ attitudes towards the NN and the help she offered did not change when interviewed three months after inclusion. Our results were found among Danish female patients with suspected or diagnosed gynecological cancer in the diagnostic period and while awaiting primary treatment. The NN was female too, which could have been of importance for the results, for instance that participants expected a kind of sister solidarity, but nobody mentioned this. What still remains new knowledge is that this study points to a connection between lack of trust in physicians before contact to an NN and/or no healthcare professional among the closest to them *and* using help from an NN outside the outpatient clinic. This is a qualitative study, and from this no causal generalizations can be made. Still, it is tempting to question whether the use of NNs in the healthcare system is nothing but a stopgap measure of a system which is not functioning optimally. However, from the physicians’ side trying to live up to all patients’ ideas of how a good physician should be might be too ambitious a task, even though physicians’ attempts to do so seems like it could contribute to a better healthcare system. It is not possible to honour all patients’ expectations, for instance in case of vulnerable patients who want surgery for cancer but are regarded inoperable. From an ethical perspective additional help should therefore probably continue to be an offer considering that the NN could be the first person to tell the patient about a suspicion of cancer.

The field of using or not using help when (suspected) sick with cancer can be complex, as shown in research on lay navigators where some patients declined navigation, but agreed with the staff to be monitored [50]. The women in our study were not offered a new NN, if they could not use the help from the contacting NN, and it could be questioned whether the non-use could be related to something personal about the NN. This cannot be refuted, but on the other hand it must be emphasized that in this study the non-users all made spontaneous, clear statements about their reason not to use the NN – they had another better help before the NN contacted them.

The NN took the primary contact to the female patients and offered help in the early part of a cancer trajectory. The NN was thereafter available for contact unless they made a plan where the NN should continue to have the proactive role in the contact. Moreover, both the female cancer patients who could use the extra help from the NN and the female cancer patients who could not presented differences at least with regard to ages, marital status, place of residence, experienced diagnostic phase, (suspicion of) cancer diagnosis (Table 1), and experienced financial situation, time from first experienced symptom to referral to the university hospital, as well as bad experiences with nurses before talking to the NN. Furthermore, some considered healthcare professionals to be very great authorities, others did not. It is in this kind of setting the results from this study might primarily be transferable. In studies from the US and Canada, both men and women were offered help from an NN, but nothing was mentioned regarding sexual differences and why some patients could not use the help on offer [15,16]. It is therefore possible the results might be transferable to a wider range of settings, but this would call for specific investigations, where focus is more on gender and possibly other diseases.

## Conclusions

In an outpatient period prior to initial treatment of cancer all participants could benefit from feeling confident of receiving help from a healthcare professional, when needed, in their cancer trajectory. Here physicians seemed to be of special importance. Trust or lack of same in the physicians and the health care system expressed by the patients was found to influence how they interrelated with the Nurse Navigator (NN). This paper specifically adds the point that previously established guarded trust (or even distrust) of physicians, combined with no caring healthcare professional among those closest to them, might be one of eventually more possible triggers of choosing additional help from an NN. As patients’ maintenance or creation of trust interacts with the way healthcare professional act verbally and nonverbally, these findings put forth the question of the importance of healthcare professionals being culturally sensitive when interacting with the patients. Not all patients could use the additional help from the NN. This is vital knowledge for healthcare practitioners as well as for administrators, as many wish to do their best for cancer patients but are obliged to consider financial implications. How we can most effectively identify those who can be helped by offering an NN is still an open question and may be focus for further debate in the healthcare system and focus for further research.

## Abbreviations

NN, Nurse Navigator.

## Competing interests

The authors declare no competing interests, financial or non-financial.

## Authors’ contributions

All authors participated in creating the idea for the study and participated in carrying out the study design. MKT carried out the study and the analysis. All authors, but primarily MKT, BDP and JK discussed interim results in the process of the analysis. MKT made the first draft of the manuscript. After a critical reading and adjustment all authors approved the final manuscript.

## Pre-publication history

The pre-publication history for this paper can be accessed here:

http://www.biomedcentral.com/1472-6963/12/168/prepub
